# The relationship between anxiety, coping strategies and characteristics of patients with diabetes

**DOI:** 10.1186/1477-7525-6-79

**Published:** 2008-10-13

**Authors:** Tarik Tuncay, Ilgen Musabak, Deniz Engin Gok, Mustafa Kutlu

**Affiliations:** 1 Faculty of Economics and Administrative Sciences, Department of Social Work, Hacettepe University, Fatih Cd. No.195 PK.06290 Kecioren – Ankara, Turkey; 2 Department of Social Services, Gülhane Hospital, Etlik – Ankara, Turkey; 3 Department of Endocrinology, Gülhane Hospital, Etlik – Ankara, Turkey

## Abstract

**Background:**

This study provided essential information, about Turkish patients with type I and type II diabetes, concerning: levels of anxiety, coping strategies used, and relationships that exist among anxiety, coping strategies, sociodemographic and medical characteristics.

**Methods:**

A sample comprising 161 Turkish adults with both types of diabetes participated in the study. The trait anxiety scale, the brief COPE, sociodemographic and medical questionnaire were administered to patients with diabetes.

**Results:**

The mean age was 49.01 (SD = 9.74), with a range from 20 to 60 years. The majority of the participants were female (60.9%) and type II diabetes (75.8%). 79% of the participants experienced anxiety. A clear majority of the participants reported to integrate their diabetes. Acceptance, religion, planning, positive reframing, instrumental support, emotional support, self-distraction and venting were the most frequently used coping strategies. The most frequently used problem-focused and the emotion-focused coping strategies were found to be similar in both type I and type II diabetes. However, participants with type II diabetes had relatively higher scores on the problem-focused strategies than those with type I. Participants with type I diabetes used humour, venting and self-blame more than those with type II diabetes. Other findings indicated that only a small minority responded to diabetes-related problems by denial, behavioural disengagement and substance use. Significant correlations were found among anxiety, coping strategies and sociodemographic characteristics of the participants. Moreover, Self-blame was found to be correlated significantly with both the problem-focused and emotion-focused coping strategies. Self-blame was also significantly correlated with both instrumental support and emotional support indicated that higher self-blame caused more frequent use of instrumental and emotional support by patients with diabetes.

**Conclusion:**

The findings of this study indicate that care for patients with diabetes should address their physical, psychological, social and economic wellbeing and the findings point to the importance of taking individual coping strategies into account when evaluating the impact of diabetes on psychosocial wellbeing. Because of the mean of anxiety were not in normal range, for this study, health professionals need to pay attention to patient's psychological state. This is especially true for patients who are likely to use self-blame and behavioural disengagement as a coping strategy. Through psychosocial interventions, professionals need to assist patients in establishing positive self evaluations. Delineation of coping strategies might be useful for identifying patients in need of particular counselling and support.

## Background

Medical advances throughout the 20th century have resulted in the transformation of many acute and once incurable illnesses into chronic conditions. As a result, the prevalence of chronic diseases, and the prevalence of diabetes in particular, has increased rapidly. In addition, environmental factors such as pollutants, and lifestyle changes such as sedentary habits and overeating have also contributed to the rise in chronic illnesses. Consequently, diabetes is one of the most challenging and burdensome chronic diseases of the 21st century, and it is a growing threat to the world's public health [1,2]. The treatments for diabetes and its associated health-risk factors are often highly complex and require considerable patient education and frequent medical monitoring [3]. At the same time, diabetes carries with it a considerable amount of stress. People on insulin must learn how to regulate their blood sugars by monitoring blood glucose levels daily while carefully attending to their food intake and an exercise regimen. Careful blood glucose monitoring is necessary to prevent wide variations in blood sugars that affect both short term and long term health and functioning. Hypoglycemic reactions are a concern in the short run not only because they are frightening and disruptive, but also because, when severe, they can lead to unconsciousness, coma and death [4].

The constant stress of maintaining tight glycemic control can result in two types of psychological distress (a) subclinical emotional distress, and (b) diagnosable psychological disorders [5]. Additionally, psychiatric conditions can occur independently without being a consequence of diabetes. It has been shown that individuals with diabetes have a disproportionately higher rate of psychiatric disorders [6,7], with affective and anxiety disorders being more commonly diagnosed than in the general population [8]. This is evidenced by research showing high rates of psychiatric disorders, particularly depression and anxiety, in a sample of Turkish patients with diabetes. For example, Fettahoglu et al., [9] found over 40% increased risk in having any type of psychiatric disorder in patients with diabetes, and Gülseren et al. [10] found that depression and anxiety account for 45% of psychiatric disorders in patients with diabetes. These results show the negative impact that diabetes can have on an individual's psychosocial adjustment, and the need for research to determine the most appropriate and common coping strategies to deal with the stress of illness.

The coping strategies used to deal with diabetes can play a key role in the maintenance and duration of, and psychosocial adjustment to diabetes [11-13]. In terms of which coping strategies are used to deal with diabetes, there is much debate as to whether the individual's appraisal of the illness as controllable or uncontrollable plays a role in choice of coping strategy and in the outcomes associated with the illness. In response to these issues, this study investigated diabetes-related coping strategies and their relationship to anxiety and sociodemographic characteristics of patients with type I and II diabetes.

### Coping

Coping has been defined as a response aimed at diminishing the physical, emotional and psychological burden that is linked to stressful life events and daily hassles [14]. Coping is understood to be adaptational activity that involves effort. It is the element of effort which enables us to draw the distinction between coping and ready-made adaptational devices such as reflexes. Coping constitutes constantly changing cognitive, behavioural and emotional efforts to manage particular external and/or internal demands that are appraised as taxing or exceeding the resources of the individual [15].

Essentially, coping strategies are separated into emotion-focused and problem-focused. An emotion-focused strategy emphasizes that patients try to process their emotions by acting and thinking. When patients use a problem-focused strategy, they believe that they can affect the situation that was caused by their disease or affect their resources to manage the situation, and this type of strategy is important to maintain quality of life. Emotion-focused and problem-focused coping strategies may be used simultaneously or alternately. It is therefore difficult to discriminate between them in the coping process [16-18]. The outcome of the coping process is adaptation or maladaptation. Adaptation is defined as the degree to which patients cope psychologically, socially and physiologically with their chronic illness [19].

Coping with the implications of one's diabetes related problems could be a difficult and often lifelong process. Patients may cope by adjusting their social role to fit the demands and challenges associated with the illness, or they may cope by trying to reframe their experiences viewing the situation in a more positive light. Accepting the reality of the diagnosis and developing a positive attitude toward treatment is thought to be critical for successful coping and recovery [20].

Coping is considered one of the core concepts in health psychology and in the context of quality of life, and is strongly associated with the regulation of emotions throughout the stress period [21]. But there is no consensus as to which coping strategies are most effective, and how well a coping strategy serves the purpose of solving problems, relieving emotional distress. However, previous research has shown that emotion-oriented coping strategies in the long run may be less adaptive than problem-oriented strategies, although the impact of these coping strategies appears to depend on the specific constraints imposed by the stressful situation [22,23]. While this is true, it has also been suggested that use of problem-focused or emotion-focused strategies might be dependent on the nature of the illness. For example, it has been suggested that persons with chronic illnesses such as myalgic encephalomyelitis/chronic fatigue syndrome (ME/CFS) with no definite cure may ascribe their illness to uncontrollable factors and therefore tend to use more emotion-focused coping strategies [24,25]. Therefore, it is possible that when an illness is associated with controllable factors, individuals with the illness engage in problem-focused solutions but when the illness is not amenable to cure or factors that are controllable, the tendency to use more emotion-focused strategies may emerge. Based on this premise, it is expected that persons with a chronic illness such as diabetes may use both problem-focused and emotion-focused strategies. Whereas problem-focused strategies might be used to better manage the physical need to monitor and administer insulin as needed and also maintain a healthy diet, the emotion-focused strategies might be evoked by the stress associated with knowing that there is currently no cure for diabetes.

In conclusion, coping strategies are related with the regulation of emotion, especially anxiety, throughout the illness process of patients with diabetes, and many studies have shown that problem-focused coping strategies are associated with less anxiety, while emotion-focused ones are associated with more anxiety [21,23,26]. However, the adaptive qualities of various coping strategies must be evaluated in the specific context where they occur.

Because of the lack of community health care in Turkey, the psychological state and coping strategies of patients with diabetes are unclear and need to be identified. However, no relevant studies in Turkey could be located. To address this gap of knowledge, the objectives of this study were to explore and describe in Turkish patients with type I and type II diabetes: (1) levels of anxiety, (2) coping strategies used, and (3) relationships among medical, sociodemographic characteristics, anxiety and coping strategies.

## Methods

### Participants and procedures

The convenience sampling method was used in this study. Patients with disease duration of less than six months and with severe physical co-morbidity were excluded from the study. Each participant was informed, prior to the interview, about the purpose of the study, written informed consent was obtained, and participants were told that they had the right to refuse participation and could withdraw at any time. One hundred and eighty-nine (189) people with diabetes completed the questionnaire. Out of these, twenty-eight (28) participants were excluded from the study due to poor response quality. Those excluded were mainly older people (mean age of 54.1 years), reporting low level of education, and had not given response to at least 70% of items of the scales. Finally, One hundred and sixty-one (161) patients (98 female, 63 male) participated in the study. Written informed consent was approved by the Gülhane Hospital's ethical review board and obtained from each participant prior to the interview (See Table 1).

**Table 1:** Sociodemographic and medical characteristics of the participants (n = 161).

**Variables**	**n (%)**
*Gender*	
Female	98 (60.9)
Male	63 (39.1)
*Age (years) mean (SD); range*	*49.01 (9.74); 20–60*
*Marital status*	
Single	11 (6.8)
Married	130 (80.7)
Widowed	20 (12.5)
*Educational status*	
Primary school	71 (44.1)
Secondary school	14 (8.7)
High School	39 (24.2)
University (undergraduate degree)	37 (23.0)
*Monthly income (New Turkish Lira-YTL)*	
Up to 500	21(13.1)
501–1000	57 (35.4)
1001–1500	23 (14.3)
1501–2000	35 (21.7)
Over 2001	25 (15.5)
*Family Type*	
Nuclear	148 (91.9)
Extended	13 (8.1)
*Employment status*	
Employed	23 (14.3)
Unemployed	138 (85.7)
*Type of settlement*	
Flat	128 (79.5)
Slums	33 (20.5)
*Type of Diabetes*	
Type 1	39 (24.2)
Type 2	122 (75.8)
*Duration of Diabetes (years) mean (SD); range*	*10.17(7.2); 1–32*

Sociodemographic and medical characteristics of the participants are presented in Table 1 (*n *= 161). The mean age of the participants with diabetes was 49.01 (SD = 9.74), with a range from 20 to 60 years, and their mean duration of diabetes was 10.17 (SD = 7.2). All of the patients were recruited from the diabetes clinic of the Gülhane Hospital. The majority of the participants were female (60.9%), married (80.7%), living in a nuclear family system (91.9%), unemployed (85.7%), living in a flat (79.5%) (middle-class family), and type II diabetes (75.8%). The distribution of educational status was: (1) primary school level, 44.1%, (2) secondary school level, 8.7%, (3) high school level, 24.2%, and (4) university level, 23.0%.

### Instruments

Anxiety was assessed using the trait scale of the Spielberger State-Trait Anxiety Scale. This is a well-validated, twenty-item, four-option response format instrument, with scale scores adjusted to fall between 20 and 80. Previous studies have identified a normative score of 25, and a clinically significant score of 42. Acceptable validity and reliability has been reported in various populations [27,28]. The validity and reliability of this inventory for Turkish society were studied by Öner and LeCompte [29].

Carver, Scheier, and Weintraub [16] developed the COPE as a comprehensive questionnaire of 15 theoretically derived coping styles or strategies. An abbreviated version of the COPE has been developed – the brief COPE [30]. The scale was administered to assess patients' coping strategies. In health psychology, the COPE and the brief COPE have predicted clinically relevant outcome across many stressful situations and populations [e.g., [16,30-33]]. The brief COPE Scale is a 28-item self report measure of problem-focused versus emotion-focused coping skills. The scale consists of 14 domains/sub-scales (self-distraction, active coping, denial, substance use, use of emotional support, use of instrumental support, behavioural disengagement, venting, positive reframing, planning, humour, acceptance, religion, self-blame) of two items each. Participants are asked to respond to each item on a four-point Likert scale, indicating what they generally do and feel when they experience diabetes-related stressful events (1 = I have not been doing this at all – 4 = I have been doing this a lot). The higher the score on each coping strategy, the greater the use of the specific coping strategy.

The brief COPE scale has good internal consistency and test-retest reliability, and concurrent validity has been established. The validity and reliability of this inventory for Turkish society were studied by Tuna [34]. In this study, Cronbach's Alpha of the brief COPE Scale was found to be .82. With regard to the internal consistency of the fourteen sub-scales for assessing coping strategies, the following Cronbach's alphas were found: acceptance .82, religion .77, planning .75, positive reframing .87, using instrumental support .76, active coping .83, using emotional support .71, humour .89, self-distraction .73, venting .84, self-blame .92, behavioural disengagement .81, denial .96, and substance use .92. The scales are only two items each, their reliabilities all meet or exceeded the value of .50 regarded as minimally acceptance [16,35].

The researchers developed a sociodemographic questionnaire including gender (1 = female; 2 = male), age, marital status, educational status, monthly income, family type, employment status, type of settlement, and type and duration of diabetes. These variables were implemented as control variables. Type and duration of diabetes were determined by self-report, asking whether the participants have type I or II diabetes. Self-reports of diabetes type were validated against the patients' medical records from the diabetes clinic. The data were consistent.

All data were collected by two of the researchers between June 2007 and February 2008 in the diabetes clinic of Gülhane Hospital. The researchers obtained permission to collect data, from the ethical review board of the Gülhane Hospital.

### Statistical analyses

The analyses were conducted using the SPSS program version 14.0. Statistical analyses included descriptive statistics, reliability testing, and Pearson product moment correlation among variables. In descriptive statistics, proportion is used to describe categorical and numerical variables; mean and SD are used to describe continuous variables. In association analyses, Pearson correlation coefficients are calculated to examine the relationships among the all variables. Levels of significance are indicated at both .05 and .01 in the correlation table. Internal consistency of the brief COPE scale was estimated using Cronbach's alpha coefficients. Participants who had given response to at least 70% of items included in any scale were included in the study.

## Results

The total mean and standard deviation of trait anxiety in two types of diabetes was 46.98 ± 6.14. The mean of trait anxiety score of patients with type I diabetes was found to be a relatively higher (48.61 ± 5.20) than those with type II diabetes (46.46 ± 6.35). 79% (n = 127) of the participants exceeded a trait anxiety threshold score of 42.

The most used problem-focused coping strategies in both type I and type II diabetes included (table 2): acceptance (7.22 ± 1.07), religion (7.07 ± 1.31), planning (6.77 ± 1.07), positive reframing (6.55 ± 1.25), using instrumental support (6.47 ± 1.62), active coping (6.15 ± 1.61), and using emotional support (5.94 ± 1.64). The most used emotional coping strategies were self-distraction (6.36 ± 1.43) and venting (5.35 ± 1.20). The most frequently used problem-focused and the emotion-focused coping strategies were found to be similar in both type I and type II diabetes. However, participants with type II diabetes had relatively higher scores on the problem-focused strategies than those with type I diabetes (see Table 2).

**Table 2:** Means and standard deviations of trait anxiety and coping strategies of the participants of two types of diabetes (n = 161).

	**Type I Diabetes (n = 39)**		**Type II Diabetes (n = 122)**		**Total (n = 161)**	
**Variables**	**M (SD)**	**Range**	**M (SD)**	**Range**	**M (SD)**	**Range**

*Trait Anxiety*	48.61 (5.20)	41–59	46.46 (6.35)	29–63	46.98 (6.14)	29–63
						
*Coping Strategies*						
*Problem-focused coping strategies*						
Acceptance	6.69 (1.43)	3–8	7.40 (0.87)	5–8	7.22 (1.07)	3–8
Religion	6.97 (1.26)	4–8	7.10 (1.32)	2–8	7.07 (1.31)	2–8
Planning	6.48 (0.82)	5–8	6.86 (1.13)	4–8	6.77 (1.07)	4–8
Positive Reframing	6.33 (1.13)	4–8	6.62 (1.29)	3–8	6.55 (1.25)	3–8
Using Instrumental Support	5.51 (1.73)	2–8	6.08 (1.59)	2–8	6.47 (1.62)	2–8
Active Coping	5.69 (1.68)	3–8	6.30 (1.56)	3–8	6.15 (1.61)	3–8
Using Emotional Support	5.51(1.73)	2–8	6.08 (1.59)	2–8	5.94 (1.64)	2–8
Humour	4.58 (2.04)	2–8	3.90 (1.90)	2–8	4.07 (1.95)	2–8
*Emotion-focused coping strategies*						
Self-Distraction	5.92 (1.64)	3–8	6.50 (1.33)	3–8	6.36 (1.43)	3–8
Venting	5.56 (1.12)	2–8	5.28 (1.22)	2–8	5.35 (1.20)	2–8
Self-Blame	4.69 (1.82)	2–8	4.04 (1.84)	3–8	4.20 (1.85)	2–8
Behavioural Disengagement	3.66 (1.57)	2–7	3.67 (1.62)	2–8	3.67 (1.61)	2–8
Denial	3.76 (1.51)	2–8	3.35 (1.63)	2–8	3.45 (1.60)	2–8
Substance Use	3.25 (1.66)	2–6	2.37 (1.25)	2–8	2.59 (1.41)	2–8

The correlation coefficients among sociodemographic, medical variables, anxiety and coping strategies are presented in Table 3. All tests were two-tailed and conducted at 5% significance. Coefficients of correlations between age, type of diabetes and duration of diabetes, gender, educational status and monthly income were significant, whereas the correlations between type of diabetes and gender, educational status and family type were non-significant. However, strong positive correlations were found among type of diabetes and age, whereas type of diabetes was negatively associated with educational status, indicating that people with type II diabetes in the present study are older and less educated than those with type I.

**Table 3:** Correlations among sociodemographic – medical characteristics, anxiety and coping strategies (n = 161).

**Variables**	**1**	**2**	**3**	**4**	**5**	**6**	**7**	**8**	**9**	**10**	**11**	**12**	**13**	**14**	**15**	**16**	**17**	**18**	**19**	**20**	**21**	**22**
*Sociodemographic characteristics*																						
Age (1)	1	.00	-.17*	-.12	.13	.46**	.21**	.05	.01	.09	.21**	.11	.06	.12	-.08	-.18*	.10	-.03	-.14	.05	-.01	.13
Gender (2)		1	.46**	.19*	-.00	-.02	-.13	.38**	-.02	-.08	-.10	-.10	-.08	-.29	-.09	-.10	-06	-.28**	.02	-.27**	.03	.10
Educational status (3)			1	.63**	-.07	-.13	-.15	.44**	-.00	-.24**	.06	.02	-.02	-.24**	-.24**	-.01	.01	-.20**	.01	-.29**	-.11	-.04
Monthly income (4)				1	-.13	-.08	-.03	.31**	-.05	-.30**	-.05	-.03	-.09	-.26**	-.08	.04	-.09	-.04	.01	-.23**	-.15	-.09
Family Type (5)					1	.16*	.01	-.03	.04	.00	.00	.23**	-.01	.17*	-.03	-.05	.06	.03	-.04	-.09	-.05	.21**
*Medical characteristics*																						
Type of Diabetes (6)						1	-.01	-.15	.28**	.04	.14	.09	.01	.16*	.14	-.14	.17*	-.06	-.14	.00	-.11	-.26**
Duration of Diabetes (7)							1	.03	-.04	.03	.00	.06	-.15*	-.18*	-.33**	.04	.06	-.07	-.04	-.02	-.01	.15
*Trait Anxiety *(8)								1	-.30**	-.39**	-.05	-.19*	-.13	-.05	-.29**	-.08	-.29**	-.23**	-.12	.01	.02	-.00
*Coping Strategies*																						
*Problem-focused strategies*																						
Acceptance (9)									1	.26**	.24**	.35**	.05	.02	.11	.09	.17*	-.08	.01	-.20*	-.03	-.15*
Religion (10)										1	.03	.26**	.11	.02	.24**	-.10	.21**	.09	.08	.06	.05	-.03
Planning (11)											1	.31**	.09	.32**	.04	.04	.17*	-.12	.07	-.26**	-.08	-.06
Positive Reframing (12)												1	.29**	.32**	.25**	.13	.33**	.16*	.09	-.12	-.00	-.04
Using Instrumental Support (13)													1	.14	.31**	-.13	.09	.50	.34**	.07	-.16*	.01
Active Coping (14)														1	.23**	.10	.17*	.02	-.12	.01	.14	.02

The mean trait anxiety scores were positively correlated with gender, educational status, and monthly income. Males had higher levels of anxiety than females, individuals with higher degree of education had more anxiety than those with less education and persons who have higher monthly income had higher levels of anxiety than individuals with less monthly income.

Strong significant correlations were found among the problem-focused coping strategies. These were planning, positive reframing, religion, instrumental support, active coping and emotional support. Strong significant correlations were also found among the emotion-focused coping strategies. These were self-distraction, behavioural disengagement, venting, self-blame, denial and substance use. In addition, venting correlated significantly with both problem-focused and emotion-focused coping strategies. Religion, as a problem focused coping strategy, was found to be negatively correlated with educational status and monthly income indicates that patients with lower level of education and monthly income more frequently use of religious coping strategies. Religion was also significantly correlated with positive reframing, emotional support and self-distraction.

Instrumental support was significantly correlated with emotional support, positive reframing and self-blame, and negatively correlated with denial. Emotional support were also positively correlated with religion, positive reframing, instrumental support, active coping as problem-focused coping strategies, and self-distraction, venting, self-blame, behavioural disengagement as emotion-focused coping strategies. Interestingly, self-blame was significantly correlated with both instrumental support and emotional support indicates that higher self-blame is related to more frequent use of instrumental and emotional support by patients with diabetes.

## Discussion

This study explored anxiety and dimensions of problem-focused and emotion-focused coping strategies -as measured by the brief COPE- in a sample of patients with type I and type II diabetes. Almost 79% of the participants in this study experienced anxiety related to their diabetes. This percentage of anxiety is higher than those found in previous research. Gülseren et al. [10] found that anxiety was one of the problems reported with 34.4% by patients with diabetes. These results show that while planning the treatment of patients with diabetes, evaluating their mental health might help to provide optimal treatment and psychosocial care services. In addition, participants with type I diabetes had higher mean of anxiety score than those with type II diabetes. This finding is consistent with prior findings. Grigsby et al. [36] and Cohen & Kanter [26] found that patients with type I diabetes experience more anxiety than patients with type II diabetes, because of the highly demanding and challenging regimen of diabetes and insulin dependency.

Coping theorists often emphasize the benefits of problem focused coping, such as acceptance, positive reframing, and turning to religion or spirituality [30,37]. A considerable number of research with various patient groups show that an increase in the functioning of spiritual or religious coping in the patients with diabetes decreases anxiety, depression, and hopelessness, and stimulates psychological functions, adaptation to the illness process, life satisfaction, and quality of life [e.g., [5,38,39]]. In a research with various chronic illnesses including diabetes by Rowe and Allen [39], the relationship between spirituality and coping was analyzed. A positive correlation was identified between the increase in the interpersonal and transcendental connectedness of the patients and their psychological wellbeing and functions. The present study documented some evidence for such benefits, in those patients with diabetes. The problem-focused coping strategies most frequently used, in this study, were acceptance, religion, planning, positive reframing, using instrumental support, active coping, and using emotional support. Self-distraction and venting considered emotion-focused strategies were also used. These findings also imply that most of the participants positively appraised their stressful and threatening disease and attempted to develop effective coping strategies to maintain their psychosocial wellbeing.

An encouraging finding from the present study was that the majority of the participants responded to their diabetes-related problems by problem-focused coping strategies instead of emotion-focused coping strategies such as behavioural disengagement, denial, and substance use. Although the problem-focused and the emotion-focused coping strategies were used in similar frequency by participants with both type I and type II diabetes, participants with type I diabetes used humour, venting and self-blame more than those with type II diabetes. These findings are similar with a research by Karlsen & Bru [23]. They also found that only a small minority of the patients used emotion-focused coping strategies such as denial, mental disengagement and resignation.

Results of correlation analysis showed that problem-focused coping strategies such as acceptance, religion, positive reframing, and emotional support were negatively related to anxiety. Thus, evidence was consistent with the idea that higher levels of anxiety are associated with lower problem-focused coping strategies. Anxiety was also found to be negatively correlated with venting and self-distraction as emotion-focused coping strategies. This finding indicates that lower levels of anxiety are associated with increased use of venting and self-distraction of diabetes related emotional distress. This finding is in conflict with that of other studies, in that prior findings indicate significant positive correlations between venting or self-distraction of one's emotions with adverse outcomes, such as distress and physical health symptoms [40-42]. The differences in the findings of this study and those of previous studies, regarding the effect of venting as a coping strategy on one's level of anxiety, may be indicative of cultural differences in how patients from various cultures distract or vent their diabetes related distress. The participants in this study indicated that venting was an effective way to promote psychosocial wellbeing and when someone said puzzling or distressful things, they 'let the unpleasant feelings escape' and felt relieved or comfortable. The advantage of venting was not only a means to release unpleasant feelings, but also a means to get an effective response from others. This finding suggests that health professionals should patiently listen to patients with diabetes and provide opportunities for expression of negative feelings and complaints.

Findings from the current study highlight the complex relationship that social support has with diabetes related coping. Results showed that instrumental support tended to be more strongly associated with problem-focused coping, and emotional support tended to be more strongly associated with less problem-focused coping. The present study suggests that different types of support have different effects. These differences between instrumental support and emotional support and their relations to coping and anxiety represent one of the most salient findings of this cross-sectional study. They also underscore the importance of modeling support as multidimensional, and of evaluating the role of support in the context of coping strategies.

Not surprisingly, denial was significantly correlated with behavioural disengagement and substance use, and inversely related to acceptance. Denial was also found to be negatively correlated with instrumental and emotional support. This finding supports the belief that denial is one of the passive coping strategies, while acceptance, instrumental support and emotional support are active coping strategies. Denial is used in 'an attempt to reject the reality of the stressful event' [41]. However, instrumental or emotional support consists of 'seeking assistance, information, or advice' to solve a stressful issue on the basis of appropriately assessing reality. Thus, when one uses denial as a coping strategy, he/she avoids confronting the reality of the situation, the opposite of using instrumental or emotional supports, which involves confronting the reality of the situation.

Self-blame was found to be correlated significantly with both the problem-focused and emotion-focused coping strategies. Although patients with diabetes wish to cope actively with the highly demanding regimen of diabetes, they may at the same time blame themselves too much for not achieving the demands of this regimen. Self-blame seems to be a double-edged sword. On the one hand, it may stimulate active coping, on the other hand, it may lead to guilt and even depression [23,43]. The dilemma between being active in coping with diabetes-related challenges and self-blaming should be a subject for further research.

The coping strategies of patients with type I and type II diabetes were unclear in Turkish population. This research, as the first study in Turkish sample, addressed this gap of knowledge in a wide range of age. This is the strength of our study. However, potential limitations of our study are that the number of patients participated in the study, and the statistical methods used for data analysis. The statistical methods limited the generalizability of our results. Another potential limitation is that there may be other variables predictive of anxiety and coping strategies used that were not considered in this analysis.

## Conclusion

Because of the non-random and small sample size of this study, the generalizability of the results may be limited. This study used a cross-sectional design, which investigates the real world at one point in time. Such a design does not examine longitudinal fluctuations in anxiety or coping strategies. Thus, longitudinal research is needed to examine psychosocial factors among patients with diabetes. In addition, further study is needed to investigate psychosocial interventions that decrease anxiety and facilitate useful coping strategies among patients with diabetes.

The findings of this study provided essential information, about Turkish patients with type I and type II diabetes, concerning: (1) levels of anxiety, (2) coping strategies used, and (3) relationships that exist among anxiety, coping strategies, sociodemographic and medical characteristics. The findings also suggest implications for psychosocial practice. Because of the mean of anxiety were not in normal range, for this study, health professionals need to pay attention to patient's psychological state. This is especially true for patients who are likely to use self-blame and behavioural disengagement as a coping strategy. Through psychosocial interventions, professionals need to assist patients in establishing positive self evaluations. For example, encouraging the use of venting as a coping strategy can assist in decreasing anxiety. In addition, identifying patients who are more likely to encounter difficulties dealing with the impacts of diabetes and then assisting them with the mobilization of problem-focused coping strategies can help foster good health behaviours. In conclusion, our findings point to the importance of taking individual coping strategies into account when evaluating the impact of disease on psychosocial wellbeing. Delineation of coping strategies might be useful for identifying patients in need of particular counselling and support.

## Competing interests

The authors declare that they have no competing interests.

## Authors' contributions

TT conceived of the study, participated in the study design and wrote the manuscript. TT, IM and DEG carried out the data analysis. MK participated in the study design and critically reviewed the manuscript, and all authors read and approved the final manuscript.
